# Grasp quality measures: review and performance

**DOI:** 10.1007/s10514-014-9402-3

**Published:** 2014-07-31

**Authors:** Máximo A. Roa, Raúl Suárez

**Affiliations:** 1 Institute of Robotics and Mechatronics, German Aerospace Center (DLR), 82234 Wessling, Germany; 2 Institute of Industrial and Control Engineering (IOC), Universitat Politècnica de Catalunya (UPC), 08028 Barcelona, Spain

**Keywords:** Grasping, Manipulation, Robotic hands, Grasp quality

## Abstract

The correct grasp of objects is a key aspect for the right fulfillment of a given task. Obtaining a good grasp requires algorithms to automatically determine proper contact points on the object as well as proper hand configurations, especially when dexterous manipulation is desired, and the quantification of a good grasp requires the definition of suitable grasp quality measures. This article reviews the quality measures proposed in the literature to evaluate grasp quality. The quality measures are classified into two groups according to the main aspect they evaluate: location of contact points on the object and hand configuration. The approaches that combine different measures from the two previous groups to obtain a global quality measure are also reviewed, as well as some measures related to human hand studies and grasp performance. Several examples are presented to illustrate and compare the performance of the reviewed measures.

## Introduction

Grasping and manipulation with complex grippers, such as multifingered and/or underactuated hands, is an active research area in robotics. The goal of a grasp is to achieve a desired object constraint in the presence of external disturbances (including the object’s own weight). Robot grasp synthesis is strongly related to the problems of fixture design for industrial parts (Brost and Goldberg 1996; Wang 2000) and design of cable-driven robots (Bruckmann and Pott 2013). Dexterous manipulation involves changing the object’s position with respect to the hand without any external support.

Grasp planning includes the determination of finger contact points on the object and the choice of an appropriate gripper configuration. Two approaches have been used to solve this problem (Sahbani et al. 2012; Mishra and Silver 1989): an empirical (physiological) approach, trying to mimic the behavior of the human hand (Feix et al. 2009; Cutkosky 1989), and an analytical (mechanical) approach, considering the physical and mechanical properties involved in grasping (Shimoga 1996). The empirical grasp synthesis chooses the most appropriate hand configuration for the object and task to be performed using tools such as learning by demonstration (Aleotti and Caselli 2010; Jakel et al. 2010; Kroemer et al. 2010), neural networks (Pedro et al. 2013; Leoni et al. 1998), fuzzy logic (Bowers and Lumia 2003), or knowledge-based systems (Bekey et al. 1993). Analytical grasp synthesis relies on mathematical models of the interaction between the object and the hand. It has been used for 2D polygonal (Liu 2000) and non-polygonal (Cornellà and Suárez 2005a) objects, and for 3D polyhedral objects (Ponce et al. 1997), objects based on complex surfaces (Zhu and Wang 2003) or 3D discrete objects (Liu et al. 2004c; Roa and Suárez 2009b). A recent survey on grasp planning methods for 3D objects is presented in (Sahbani et al. 2012). Grasp synthesis algorithms take into account the following basic properties:

- *Disturbance resistance*: a grasp can resist disturbances in any direction when object immobility is ensured, either by finger positions *(form closure)* or, up to a certain magnitude, by the forces applied by the fingers *(force closure)* (Bicchi 1995; Rimon and Burdick 1996). *Main problem*: determination of contact points on the object boundary.
- *Dexterity*: a grasp is dexterous if the hand can move the object in a compatible way with the task to be performed. When there are no task specifications, a grasp is considered dexterous if the hand is able to move the object in any direction (Shimoga 1996). *Main problem*: determination of hand configuration.
- *Equilibrium*: a grasp is in equilibrium when the resultant of forces and torques applied on the object (by the fingers and external disturbances) is null (Kerr and Roth 1986; Buss et al. 1996; Liu 1999; Liu et al. 2004a). *Main problem*: determination and control of the proper contact forces.
- *Stability*: a grasp is stable if any error in the object position caused by a disturbance disappears in time after the disturbance vanishes (Howard and Kumar 1996; Lin et al. 1997; Bruyninckx et al. 1998). *Main problem*: control of restitution forces when the grasp is moved away from equilibrium.

In general, given an object and a hand there is more than one grasp that fulfills a desired property; therefore, an optimal grasp is chosen using a *quality measure*, i.e. an index that quantifies the goodness of a grasp. This paper presents a review of the grasp quality measures related to disturbance resistance and dexterity, the first two properties to be considered in analytical grasp synthesis. Examples and weak and strong points in each case are also given. Most quality measures have been developed for fingertip precision grasps; the extension of these measures to underactuated and power grasps is also discussed. This work is an update and extension of the work presented by Suárez, Roa and Cornellà (Suárez et al. 2006; Roa et al. 2008).

After this introduction the article is structured as follows. Section 2 summarizes the basic background necessary to formalize the grasp quality measures. Sections 3 and 4 present the quality measures associated with the positions of contact points, and with hand configuration, respectively. Section 5 reviews the approaches that combine different measures from the two previous groups to obtain a global quality measure, and Sect. 6 presents other approaches not included in the previous groups. Finally, Sect. 7 presents the closing discussion.

## Basic background and nomenclature

### Modeling of contacts, positions, forces and velocities

The forces applied at the contact points can act only against the object (positivity constraint), and the types of contact considered between the fingertips and the object are:

- *Punctual contact without friction*: the applied force is always normal to the contact boundary.
- *Punctual contact with friction (hard contact)*: the applied force has a component normal to the contact boundary and may have another one tangential to it. Several models have been proposed to represent friction (Howe et al. 1988), the most common being Coulomb’s friction cone.
- *Soft contact*: it allows the application of the same forces as the hard contact plus a torque around the direction normal to the contact boundary. This model is valid only for 3D objects (Buss et al. 1996; Xydas and Kao 1999).

The number $r$ of independent components of the possible wrenches applied at each contact depends on the type of contact: $r=1$ for the contact point without friction, $r=2$ and $r=3$ for the hard contact in the 2D and 3D physical space, respectively, and $r=4$ for the soft contact.

A force $\varvec{F}_i$ applied on the object at a point $\varvec{p}_i$ generates a torque $\varvec{\tau }_i=\varvec{p}_i\times \varvec{F}_i$ with respect to the object’s center of mass (*CM* ). The force and the torque are grouped in a wrench vector $\varvec{\omega }_i=(\varvec{F}_i , \varvec{\tau }_i/\rho )^T$, with $\rho$ being a constant that defines the metric of the wrench space. Possible choices for this parameter include the object’s radius of gyration and the largest distance from *CM* to any point on the object’s surface. A detailed explanation of the implications of such choices can be found in Roa and Suárez (2009a). The dimension of $\varvec{\omega }$ is $d=3$ for 2D and $d=6$ for 3D objects.

The movement of the object is described through the translational velocity $\varvec{v}$ of *CM*, and the rotational velocity $\varvec{w}$ of the object with respect to *CM*. Both velocities are represented as a twist $\varvec{\dot{x}}=(\varvec{v},\varvec{w})^T\in \mathbb {R}^d$.

The force $\varvec{f}_i$ at the fingertip $i$ is produced by torques $\varvec{T}_{ij}$, $j=1,...,m$, applied at each one of the $m$ joints. In a hand with $n$ fingers, a vector $\varvec{T}=\left[ \varvec{T}_{1j}^T \ldots \varvec{T}_{nj}^T \right] ^T\in \mathbb {R}^{nm}$ is defined to group all the torques applied at the hand joints. The velocities in the finger joints, $\varvec{\dot{\theta }}_{ij}$, are also grouped in a single vector $\varvec{\dot{\theta }}=\left[ \varvec{\dot{\theta }}_{1j}^T \ldots \varvec{\dot{\theta }}_{nj}^T \right] ^T\in \mathbb {R}^{nm}$.

Forces and velocities at all fingertips can be expressed in a local reference system. Thus, the vector $\varvec{f}\!=\!\left[ \varvec{f}_{1k}^T \ldots \varvec{f}_{nk}^T \right] ^T\in \mathbb {R}^{nr}$ ($k=1,...,r$) groups all the force components applied at the contact points, and the vector $\varvec{\nu }=\left[ \varvec{\nu }_{1k}^T \ldots \varvec{\nu }_{nk}^T \right] ^T\in \mathbb {R}^{nr}$ contains all the velocity components at the fingertips.

### Relations between forces and velocities

**Fig. 1:**
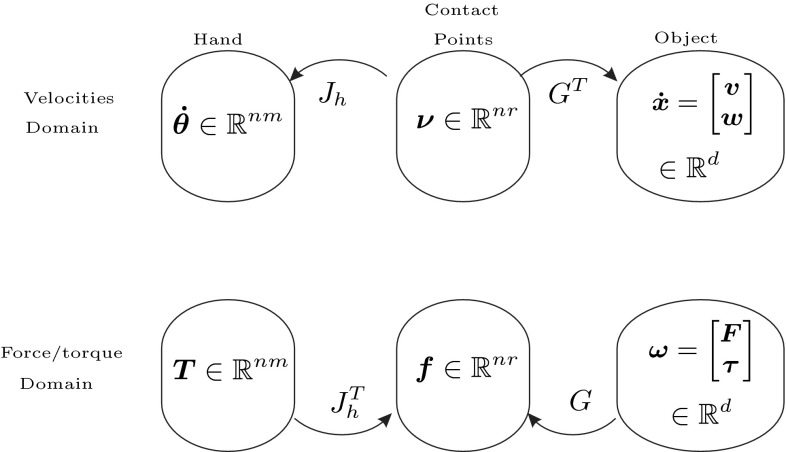
Relations between grasp force and velocity domains

Forces and velocities associated with the object, the hand and the contact points satisfy the following relations, illustrated in Fig. 1 (Murray et al. 1994):

Forces $\varvec{f}$ and velocities $\varvec{\nu }$ at the fingertips are related to torques $\varvec{T}$ and velocities $\varvec{\dot{\theta }}$ at the finger joints through the hand Jacobian, $J_h=\text {diag}\left[ J_1,\ldots , J_i\right] \in \mathbb {R}^{nr\times nm}$ where $J_i\in \mathbb {R}^{r\times m}$, $i=1,\ldots ,n$, is the Jacobian for finger $i$ that relates the variables at the finger joints with the variables at the fingertips:

1 $$\begin{aligned}&\varvec{\nu }=J_h\varvec{\dot{\theta }}\end{aligned}$$

2 $$\begin{aligned}&\varvec{T}=J_h^T\varvec{f} \end{aligned}$$

The relation between forces $\varvec{f}$ at the fingertips and the total wrench $\varvec{\omega }$ applied on the object, and the relation between velocities $\varvec{\nu }$ at the contact points and the twist $\varvec{\dot{x}}$ is given by the grasp matrix $G\in \mathbb {R}^{d\times nr}$:

3 $$\begin{aligned} \varvec{\nu }=G^T\varvec{\dot{x}}\end{aligned}$$

4 $$\begin{aligned} \varvec{\omega }=G\varvec{f} \end{aligned}$$

Note that from (1) to (3), the *fundamental grasping constraint* that relates velocities of the finger joints to velocities of the object can be obtained (Murray et al. 1994):

5 $$\begin{aligned} J_h\varvec{\dot{\theta }}=G^T\varvec{\dot{x}} \end{aligned}$$

Using (3), it is also possible to obtain the object’s velocity starting with the velocities at the contact points:

6 $$\begin{aligned} \varvec{\dot{x}}=(G^T)^+\varvec{\nu } +N(G^T)\varvec{\nu }_0 \end{aligned}$$

where $(G^T)^+$ denotes the pseudoinverse of $G^T$, $N(G^T)$ is a matrix whose columns form a basis for the null space of $G^T,\, \mathcal{N}(G^T)$, and $\varvec{\nu }_0$ is an arbitrary vector that parametrizes the solution set. The pseudoinverse is required as $G^T\in \mathbb {R}^{nr\times d}$ is generally not a square matrix^1^. To produce any twist or wrench on the object, it is required that $\mathcal{N}(G^T)=\varvec{0}$, or equivalently, that $rank(G)=d$ (Prattichizzo and Trinkle 2008). This condition further simplifies (6) to $\varvec{\dot{x}}=(G^T)^+\varvec{\nu }$.

The direct transformation in the velocity domain from the higher dimensional hand joint space to the lower dimensional object space can then be obtained via the hand-object Jacobian $H$ as

7 $$\begin{aligned} \varvec{\dot{x}}=H\varvec{\dot{\theta }} \end{aligned}$$

where $$H=(G^T)^+J_h\in \mathbb {R}^{d\times nm}$$.

Note that the above analysis relies on a quasi-static approach, as dynamics is not typically considered to play a major role in grasping tasks, although interesting dynamic grasping and manipulation behaviors have been reported (Senoo et al. 2009). Also, it is assumed that every finger has full mobility in its task space, which is not true for *defective systems*, i.e. systems that have links with limited mobility, such as the palm in a hand that performs a power grasp. For these systems, specific solutions to the problem of distributing perturbation forces to the contact points can be obtained (Bicchi 1994).

## Quality measures associated with the position of contact points

This first group of quality measures includes those that only take into account the object’s properties (shape, size, weight), friction constraints and form and force closure conditions to quantify grasp quality. These measures are classified into three subgroups: one considering only algebraic properties of the grasp matrix $G$, another one considering geometric relations in the grasp (assuming in both subgroups that fingers can apply forces without a magnitude limit), and a third subgroup of measures that considers limits in the magnitudes of the finger forces.

### Measures based on algebraic properties of the grasp matrix $G$

#### Minimum singular value of $G$

A full-rank grasp matrix $G\in \mathbb {R}^{6\times r}$ has 6 singular values given by the positive square roots of the eigenvalues of $GG^T$. When a grasp is in a singular configuration, at least one of the singular values of $G$ goes to zero, and the grasp loses the capability of withstanding external wrenches in at least one direction. The smallest singular value of the grasp matrix $G$, $\sigma _{min}(G)$, is a quality measure that indicates how far the grasp configuration is from falling into a singular configuration (Li and Sastry 1988), i.e.

8 $$\begin{aligned} Q_{\tiny MSV}=\sigma _{\min }(G) \end{aligned}$$

A large $\sigma _{\min }(G)$ leads to a better grasp. Similarly, a large $\sigma _{\min }(G)$ results in larger minimum contributions (transmission gain) from forces $\varvec{f}_i$ at the contact points to the net wrench $\varvec{\omega }$ on the object, which is also used as a grasp optimization criterion (Kim et al. 2001).

$Q_{\tiny MSV}$ indicates a physical condition that may be critical in a grasp from a practical point of view. However, it is not invariant under a change in the reference system used to compute torques.

#### Volume of the ellipsoid in the wrench space

The effect of the grasp matrix $G$ on the relations given by Eq. (4) can be visualized as follows. Equation (4) maps a sphere of unitary radius in the force domain of the contact points (i.e. the set $\parallel f \parallel = 1$) into an ellipsoid in the wrench space. The global contribution of all the contact forces can be considered using the volume of this ellipsoid as the quality measure (Li and Sastry 1988), i.e.

9 $$\begin{aligned} Q_{\tiny VEW}= \sqrt{\hbox {det}{\left( GG^T\right) }}= \sigma _1\sigma _2\ldots \sigma _d \end{aligned}$$

with $\sigma _1$, $\sigma _2$,$\ldots$, $\sigma _d$ denoting the singular values of the grasp matrix $G$. Unlike the previous measure, this one considers all the singular values with the same weight and must be maximized to obtain the optimum grasp.

$Q_{\tiny VEW}$ is invariant under a change in the torque reference system, but it does not provide information about whether some fingers are contributing more than others to the grasp.

#### Grasp isotropy index

This criterion looks for a uniform contribution of the contact forces to the total wrench applied on the object, i.e. it tries to obtain an isotropic grasp where each applied contact force contributes to the object’s internal forces in a similar way. The quality measure is defined as,

10 $$\begin{aligned} Q_{\tiny GII}=\frac{\sigma _{\min }(G)}{\sigma _{\max }(G)} \end{aligned}$$

with $\sigma _{\max }(G)$ and $\sigma _{\min }(G)$ being the maximum and minimum singular values of $G$, respectively (Kim et al. 2001). This index approaches 1 when the grasp is isotropic (optimal case), and falls to zero when the grasp is close to a singular configuration.

$Q_{\tiny GII}$ indicates whether the grasp has an equivalent behavior in any direction, which may be useful for general purpose grasps; it also indirectly indicates the same physical condition as $Q_{\tiny MSV}$.

### Measures based on geometric relations

#### Shape of the grasp polygon

**Fig. 2:**
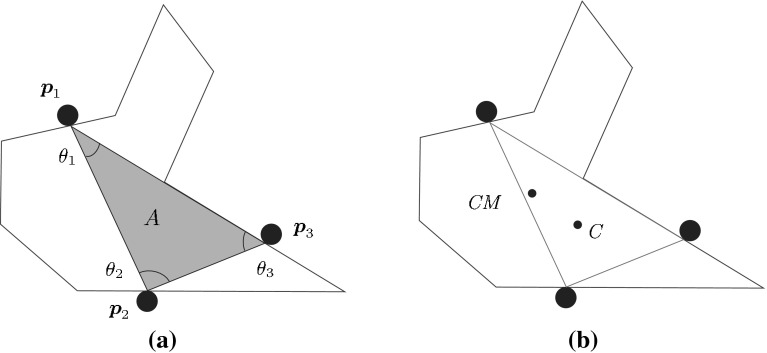
Examples of physical interpretation of quality measures based on geometric relations: **a** Shape of the grasp polygon ($Q_{\tiny SGP}$) determined by the internal angles, and area of the grasp polygon ($Q_{\tiny AGP}$); **b** Distance between the centroid $C$ of the grasp polygon and the object’s center of mass *CM* ($Q_{\tiny DCC}$)

In planar grasps (i.e. grasps with coplanar contact points, even on 3D objects) it is desirable that the contact points are uniformly distributed over the object surface to improve grasp stability (Park and Starr 1992; Mirtich and Canny 1994). An index to quantify distribution uniformity compares the distance from the internal angles of the grasp polygon defined by the contact points on the object (as illustrated in Fig. 2a) to those of the corresponding regular polygon (Kim et al. 2001). The index is

11 $$\begin{aligned} Q_{\tiny SGP}=\frac{1}{\theta _{\max }}\sum \limits _{i=1}^{n}\left| \theta _i-\bar{\theta }\right| \end{aligned}$$

where $n$ is the number of fingers, $\theta _i$ the internal angle at vertex $i$ of the contact polygon, $\bar{\theta }$ is the average internal angle of the corresponding regular polygon (given in degrees by $\bar{\theta }={180(n-2)}/{n}$), and $\theta _{\max }=(n-2)(180-\bar{\theta })+2\bar{\theta }$ is the sum of the internal angles when the polygon has the most ill conditioned shape (i.e. when the polygon degenerates into a line and the internal angles are either 0 or $\pi$). The quality index is minimum (optimum) when the contact polygon is regular (Park and Starr 1992).

$Q_{\tiny SGP}$ has a simple physical interpretation and an easy computation, but it is useful for planar grasps only. The extension to general 3D grasps is not evident, and there may be cases where $Q_{\tiny SGP}$ leads to unexpected grasps from the practical point of view (for instance grasping an elongated object, like a pencil) because of the object’s geometry.

#### Area of the grasp polygon

In 3-finger grasps, a larger triangle formed by the contact points $\varvec{p}_1,\,\varvec{p}_2$ and $\varvec{p}_3$ on the object (Fig. 2a) gives a more robust grasp, i.e. with the same finger forces the grasp can resist larger external torques (Mirtich and Canny 1994; Chinellato et al. 2003). Thus, the area of the grasp triangle is also used as a quality measure (both for 2D and 3D objects), i.e.

12 $$\begin{aligned} Q_{\tiny AGP}=\hbox {Area}(\hbox {Triangle}(\varvec{p}_1,\varvec{p}_2,\varvec{p}_3)) \end{aligned}$$

$Q_{\tiny AGP}$ has a simple physical interpretation and an easy computation as well. In theory, this index could be extended to grasps of 3D objects involving more than 3 fingers by maximizing the volume of the convex hull of the contact points; however, this has recently been shown to be non representative for grasp analysis (Roa et al. 2012; Balasubramanian et al. 2012). A useful way for getting such extension is by choosing three fingers for defining a contact plane, projecting the remaining contacts to this contact plane and then maximizing the area of the grasp polygon (Supuk et al. 2005):

13 $$\begin{aligned} Q_{\tiny AGP^\prime }=\hbox {Area}(\hbox {Polygon}(\varvec{p}_1,\varvec{p}_2,\varvec{p}_3,\varvec{p}_{P4},...,\varvec{p}_{Pn})) \end{aligned}$$

where the subindex $P$ indicates the projected contact points. Nevertheless, like $Q_{\tiny SGP}$ and $Q_{\tiny AGP}$, $Q_{\tiny AGP^\prime }$ may lead sometimes to non practical grasps. In practice, these measures should be complemented by other measures more directly related to grasp properties.

#### Distance between the centroid of the contact polygon and the object’s center of mass

The effect of inertial and gravitational forces on the grasp is minimized when the distance between the object’s center of mass, *CM*, and the centroid $C$ of the contact polygon (for 2D objects) or polyhedron (for 3D objects) is minimized (Fig. 2b). Then, this distance is also used as a grasp quality measure, both for 2D (Chinellato et al. 2005) and 3D objects (Ponce et al. 1997; Ding et al. 2001), i.e.

14 $$\begin{aligned} Q_{\tiny DCC}= \hbox {Dist}\left( CM,C\right) \end{aligned}$$

$Q_{\tiny DCC}$ has a simple physical interpretation and an easy computation only if *CM* is known, but in practice it might be difficult to know *CM* for some real objects (the object’s density is usually unknown, and even when it can be considered constant the object’s complete shape could also be partially unknown or too complex for an easy computation of *CM*). Another disadvantage that limits the applicability of this measure is that the number of contact points does not influence the quality value.

#### Orthogonality

It has recently been shown that humans tend to align their hands with the main axis of inertia of the object to be grasped (Balasubramanian et al. 2010). Let $\varvec{z}$ be the vector perpendicular to the palm surface, and $\varvec{u}$ a vector along the direction of the object’s principal axis of inertia; the angle between both vectors is computed as $\delta =\text {arccos}(\varvec{z}\cdot \varvec{u})$, and the measure is

15 $$\begin{aligned} Q_{\tiny O}= {\left\{ \begin{array}{ll} \delta , &{} \text {if}\, \delta <\pi /4,\\ \pi /2-\delta , &{} \text {if}\, \pi /4<\delta <\pi /2,\\ \delta -\pi /2, &{} \text {if}\,\pi /2<\delta <3\pi /4,\\ \pi -\delta , &{} \text {if}\,\delta >3\pi /4.\\ \end{array}\right. } \end{aligned}$$

The maximum possible value for the measure is $\pi /4$, and the minimum value is $0$. As most of the objects that humans (and robots) interact with have been designed with Cartesian coordinate frames, it seems natural that grasps are better when the palm (wrist) orientation is parallel or perpendicular to the object’s main axis of inertia, i.e. when $Q_{\tiny O}$ is close to zero. Perpendicularity of $\varvec{z}$ with respect to the ground plane has previously been used for hand preshape in a heuristic algorithm that tried to give as much leeway as possible to the hand when grasping an object (Wren and Fisher 1995).

#### Margin of uncertainty in finger positions

The space defined by the $n$ parameters representing the possible contact points of $n$ fingers on a 2D object boundary is called *grasp space* (or contact space), and the subset of the grasp space representing force closure grasps is called *force closure space*, FCS. For polygonal objects, FCS is the union of a set of convex polyhedra ${CP}_i$, and this is used in several proposals to compute the FCS for polygonal objects and any number of fingers, with or without friction (Liu 2000; Li et al. 2002; Cornellà and Suárez 2005b).

**Fig. 3:**
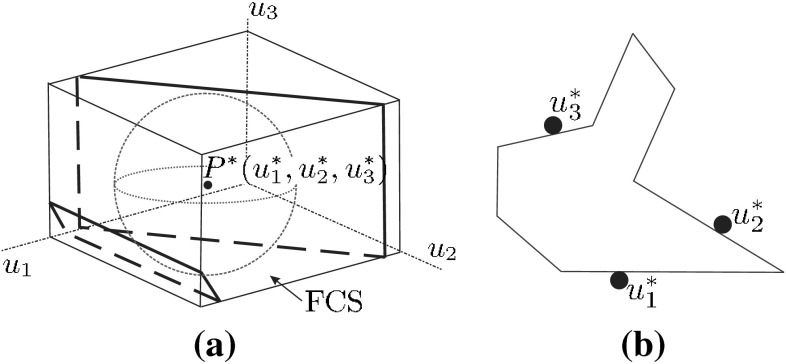
Example of the maximization of the margin of uncertainty $Q_{\tiny MUF}$ (each parameter $u_i$ fixes the position of finger $i$ on the object boundary): **a** Maximum hypersphere in the FCS centered at $P^*=(u_1^*, u_2^*,u_3^*)$ ; **b** Optimum grasp in the physical space determined by $u_1^*$, $u_2^*$ and $u_3^*$

Considering uncertainty in actual finger positioning, greater distances from the boundary of the FCS result in more secure grasps. With this criterion, given a grasp represented by a point $P$ in the grasp space, the radius of the largest hypersphere centered at $P$ and fully contained in one of the convex polyhedra ${CP}_i$ that form the FCS was proposed as a grasp quality measure, i.e.

16 $$\begin{aligned} Q_{\tiny MUF}=\min _{P_j \in \partial \, {CP}_i}\left\| P - P_j \right\| \end{aligned}$$

with $\partial \,{CP}_i$ being the boundary of ${CP}_i$. An example for 3 fingers, and therefore a 3-dimensional grasp space, is shown in Fig. 3.

$Q_{\tiny MUF}$ is quite appropriate to minimize the effect of uncertainty on finger positions during grasp execution, but it is difficult to apply to non-polygonal 2D or 3D objects due to the complexity and high dimensionality of the resulting grasp space (note that for 3D objects two parameters are needed to fix the position of each finger on the object surface).

#### Independent contact regions

The concept of *independent contact regions* refers to a set ICRS of regions ICR$_i$ on the object boundary such that a finger contact inside each ICR$_i$ produces a force closure grasp independent of the exact contact points (Nguyen 1988). The representation of the possible grasps allowed by a set ICRS is a closed region in the grasp space fully contained in the force closure space. For 2D objects and $n$ fingers, this region is an $n$-dimensional parallelepiped $B$ aligned with the reference axis. Larger regions $B$ (i.e larger edges of the parallelepiped) lead to larger sets of possible FC grasps. Also, grasping with each finger in the center of each independent contact region ICR$_i$ (i.e. in the center of $B$), results in larger positioning errors allowed for each finger. Thus, the quality of this grasp is associated with the size $L_{\min }$ of the smallest independent region ICR$_i$ (i.e. the length of the shortest edge of $B$) (Ponce and Faverjon 1995),

17 $$\begin{aligned} Q_{\tiny ICR}=L_{\min } \end{aligned}$$

$Q_{\tiny ICR}$ has a clear physical interpretation and is particularly useful in the presence of uncertainty in finger positioning. Higher quality also indicates a greater possibility of finding a set of reachable contact points allowing a force closure grasp for a given mechanical hand. As a drawback, it is necessary to compute the set ICRS (i.e. $B$), resulting in extra computational cost (Roa and Suárez 2009a).

This criterion was initially developed for polygonal objects (Nguyen 1988), and then applied to 2-finger grasps of 2D non-polygonal objects (Stam et al. 1992), producing a force closure space limited by curves. The independent regions ICRS were obtained by maximizing the area of $B$. This is a variation of $Q_{\tiny ICR}$,

18 $$\begin{aligned} Q_{\tiny ICR^\prime }= \hbox {Area}(B) \end{aligned}$$

**Fig. 4:**
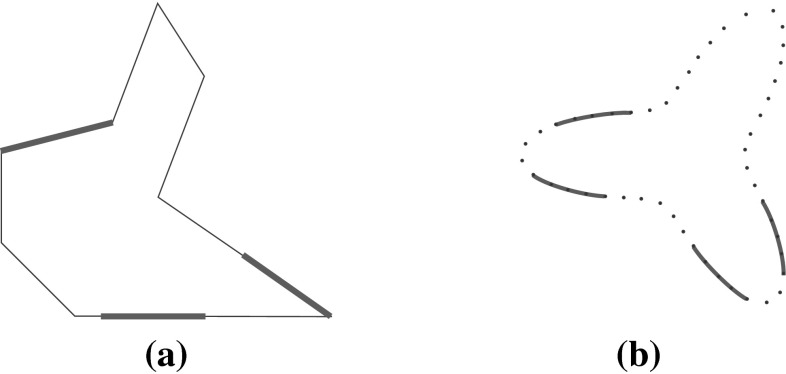
Examples of independent contact regions: **a** 3-finger grasp of a polygonal object; **b** 4-finger grasp of a non-polygonal discretized object

$Q_{\tiny ICR}$ and $Q_{\tiny ICR^\prime }$ were also adapted for 2D discretized objects of any shape (i.e. with their boundary represented by a finite number of points) (Cornellà and Suárez 2005a), with grasp quality associated with the number of points on the sides of $B$ for $Q_{\tiny ICR}$ and inside $B$ for $Q_{\tiny ICR^\prime }$. Figure 4 shows two examples of ICRS.

Another quality measure proposed for polyhedral objects and based on a set ICRS is given by the sum of the distances between each one of the $i$-th actual contact points $(x_i, y_i, z_i)$ and the center of the corresponding independent contact region $(x_{i0}, y_{i0}, z_{i0})$, i.e.

19 $$\begin{aligned} Q_{\tiny ICR^{\prime \prime }}=\frac{1}{n}\sum \limits _{i=1}^{n}\sqrt{\left( x_i-x_{i0}\right) ^2+\left( y_i-y_{i0}\right) ^2+\left( z_i-z_{i0}\right) ^2} \end{aligned}$$

$Q_{\tiny ICR^{\prime \prime }}$, also called uncertainty grasp index (Kim et al. 2001) or grasp margin (Chinellato et al. 2003), reaches the optimal value (zero) when all the fingers are located at the center of each ICR$_i$.

### Measures considering limitations on the finger forces

The previous subgroups of quality measures are related to the geometric location of contact points, but do not consider any limit in the magnitude of the forces applied by the fingers. Thus, even when the obtained force closure grasps can resist external perturbation wrenches along any direction, nothing is said about the magnitude of the perturbation that can be resisted. This means that in some cases the fingers may have to apply extremely large forces to resist small perturbations. Thus, grasp quality could also consider the module of the perturbation wrench that the grasp can resist when forces applied by fingers are limited. This section includes the quality measures that consider this aspect.

#### Largest-minimum resisted wrench

There are two common constraints on finger forces $\varvec{f}_i$. The first one is that the module of the force applied by each finger is individually limited, which corresponds to a limited independent power source (or transmission) for each finger. In order to simplify the formalism, and without loss of generality, it is assumed that all finger forces have the same limit and that it is normalized to 1, i.e. $\left\| \varvec{f}_i\right\| \le 1,\, i=1,...,n$.

By approximating the friction cone at the contact point $\varvec{p}_i$ by a pyramid with $m$ edges, the force $\varvec{f}_i$ applied by the finger can be expressed as a positive linear combination of unitary forces $\varvec{f}_{ij}$, $j=1,...,m$ along the pyramid edges (usually called primitive forces), and the wrench $\varvec{\omega }_i$ produced by $\varvec{f}_i$ at $\varvec{p}_i$ can be expressed as a positive linear combination of the wrenches $\varvec{\omega }_{ij}$ produced by $\varvec{f}_{ij}$ (primitive wrenches). Now, $n$ fingers produce a resultant wrench on the object given by

20 $$\begin{aligned} \varvec{\omega }_O&= \sum \limits _{i=1}^{n}\varvec{\omega }_i= \sum \limits _{i=1}^{n}\sum \limits _{j=1}^{m}\alpha _{ij}\varvec{\omega }_{ij}\nonumber \\&\text{ with } \alpha _{ij}\ge 0 ,\; \sum \limits _{j=1}^{m}\alpha _{ij}\le 1 \end{aligned}$$

By considering the possible variations of $\alpha _{ij}$, the set $\mathcal{P}$ of possible resultant wrenches on the object is the convex hull of the Minkowski sum of primitive wrenches $\varvec{\omega }_{ij}$:

21 $$\begin{aligned} \mathcal{P}={CH}\left( \bigoplus \limits _{i=1}^n \left\{ \varvec{\omega }_{i1},\ldots ,\varvec{\omega }_{im}\right\} \right) \end{aligned}$$

The second common constraint in the finger forces is that the sum of modules of the forces applied by $n$ fingers is limited, which corresponds to a limited common power source for all the fingers. Assuming a normalized limit of 1, the constraint is $\sum _{i=1}^n\left\| \varvec{f}_i\right\| \le 1$.

By approximating again the friction cone with a pyramid, the resultant wrench on the object is given by

22 $$\begin{aligned}&\varvec{\omega } = \sum \limits _{i=1}^{n}\sum \limits _{j=1}^{m}\alpha _{ij}\varvec{\omega }_{ij}\nonumber \\&\text{ with } \alpha _{ij}\ge 0 ,\; \sum \limits _{i=1}^{n}\sum \limits _{j=1}^{m}\alpha _{ij}\le 1 \end{aligned}$$

and now the set $\mathcal{P}$ is the convex hull of the primitive wrenches $\varvec{\omega }_{ij}$:

23 $$\begin{aligned} \mathcal{P}={CH}\left( \bigcup _{i=1}^{n}\left\{ \varvec{\omega }_{i1},\ldots ,\varvec{\omega }_{im}\right\} \right) \end{aligned}$$

The set $\mathcal{P}$ is known as *Grasp Wrench Space* GWS (Pollard 1996; Borst et al. 1999).

There are other proposals of constraints on finger forces, like $\sum _{i=1}^n\left\| \varvec{f}_i\right\| ^2\le 1$ (Mishra 1995). However, physical interpretations are not as evident as in the previous ones and have not been widely implemented.

Considering the force constraints, a grasp quality measure is defined as the largest perturbation wrench that the grasp can resist in any direction, i.e. the distance from the origin of the wrench space to the closest facet of $\mathcal{P}$ (Ferrari and Canny 1992; Kirkpatrick et al. 1992). Geometrically, the quality is equivalent to the radius of the largest ball centered at the origin of the wrench space and fully contained in $\mathcal{P}$, and therefore it is frequently referred to as the criterion of the largest ball. The quality measure is

24 $$\begin{aligned} Q_{\tiny LRW}=\min _{\varvec{\omega }\in \partial \mathcal{P}}\left\| \varvec{\omega }\right\| \end{aligned}$$

with $\partial \mathcal{P}$ being the boundary of $\mathcal{P}$. This is one of the most popular quality measures; the mathematical basis has been studied for frictionless (Mishra et al. 1987) and frictional grasps (Teichmann and Mishra 1997), and is used in several works on grasp synthesis, e.g. Borst et al. (2003); Miller and Allen (2004). An efficient method for computing it has been recently proposed (Zheng 2013).

**Fig. 5:**
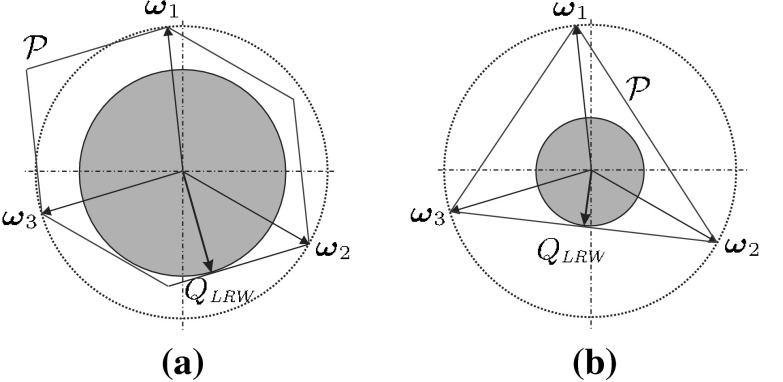
Qualitative 2-dimensional example of the grasp quality using 3 fingers and **a** a limit in the module of each force; **b** a limit in the sum of the modules of the applied forces

An optimal grasp under a force constraint is not necessarily optimal under another one. Figure 5 qualitatively illustrates the constraints on the finger forces described in Eqs. (20) and (22), the sets of possible wrenches, and the resulting qualities in each case.

The quality measure given by Eq. (24) is interpreted using the metric $L_2$. In theory, other metrics like $L_1$ or $L_\infty$ can be used (Mishra 1995). In practice, these metrics have been used for measuring grasp quality of partial force closure grasps (i.e. grasps which only immobilize the object along certain directions) by considering the sum of the needed forces applied at the existing contact points in order to exert some given unit wrench on the object (Kruger and van der Stappen 2011).

The consideration of the maximum real force that fingers can apply at each contact point is usually not taken into account. However, the real wrenches that fingers with limited torque bounds apply on the object surface, according to (2), can be used for building a set $\mathcal{P}_{r}$ that includes all the wrench space reachable by the real robot hand (Jeong and Cheong 2012; Zheng and Yamane 2013). Replacing $\mathcal{P}$ with $\mathcal{P}_{r}$ still holds for the quality measure defined in (24).

$Q_{\tiny LRW}$ has a clear and useful physical meaning for general purpose grasps, but depends on the reference system used to compute torques. Selecting the object’s center of mass as the origin of the reference system is coherent with the system dynamics, but as stated above for other measures, in some cases it may be difficult to know the center of mass accurately. Besides, it is necessary to establish a metric in the wrench space to simultaneously consider pure forces and torques, as defined by the factor $\rho$ introduced in Sect. 2.1 (Roa and Suárez 2009a). $Q_{\tiny LRW}$ can be normalized with respect to the maximum value that it can reach for a given object, which indicates how far the grasp is from being optimum. However, this requires the computation of the maximum value, implying an additional computational cost. A recent attempt to overcome the dependence of $Q_{\tiny LRW}$ on the reference frame was proposed by setting the moment origin at the centroid of contact positions so that the grasp wrench sets are frame independent (Zheng and Qian 2009). In that work, instead of dividing the torque component by a factor $\rho$ it is proposed to multiply the force components by the average distance from the contacts to their centroid, which makes that the grasp wrench sets have the same scale in all wrench directions, and sets the scale factor of the ball in the wrench space directly proportional to the same average distance.

#### Volume of the Grasp Wrench space (volume of $\mathcal{P}$)

Different alternatives have been proposed to avoid the dependence of $Q_{\tiny LRW}$ on the reference system used to compute torques, for instance using the radius of the largest ball with respect to all possible choices of reference systems as the quality measure (Teichmann 1996). However, this has not been widely considered due to its high computational cost. To deal with this problem, another alternative quality measure is the volume of $\mathcal{P}$ (Miller and Allen 1999),

25 $$\begin{aligned} Q_{\tiny VOP}=\hbox {Volume}(\mathcal{P}) \end{aligned}$$

$Q_{\tiny VOP}$ is independent of the reference system used to compute torques, but it does not indicate whether the grasp has a poor capacity of compensating perturbation wrenches in some particular directions, i.e. with the same $Q_{\tiny VOP}$ a given grasp could stand a much lower force than another one in a certain direction. As in the case of $Q_{\tiny LRW}$, it is necessary to establish a suitable metric in the wrench space to simultaneously consider pure forces and torques.

#### Decoupling forces and torques

To avoid the definition of a factor $\rho$ relating forces and torques in the wrench space, the following optimality criterion for grasp synthesis was proposed (Mirtich and Canny 1994): first, grasps that better resist pure forces are computed and, from them, grasps with the best resistance to pure torques are chosen. The quality measures used in each step are

26 $$\begin{aligned} Q_{\tiny f}&= \min _{\varvec{f}\in \partial {\mathcal {P}}^f}\left\| \varvec{f}\right\| \end{aligned}$$

27 $$\begin{aligned} Q_{\tiny \tau }&= \min _{\varvec{\tau }\in \partial {\mathcal {P}}^\tau }\left\| \varvec{\tau }\right\| \end{aligned}$$

where $\partial \mathcal{P}^f$ and $\partial \mathcal{P}^\tau$ are the boundaries of the sets of possible resultant forces and torques, respectively, that fingers can generate on the object.

$Q_{\tiny f}$ and $Q_{\tiny \tau }$ can be computed in a simpler way by avoiding the definition of a metric of the wrench space, although they are actually two independent measures and the order in which they are considered affects the solution.

#### Normal components of the forces

The sum of the components of applied forces normal to the object’s boundary is indicative of the force efficiency in the grasp. Then, a quality measure is defined as the inverse of the sum of the magnitudes of the normal components of the applied forces required to balance an expected demanding wrench $\varvec{\omega }_{ext}$ ($\varvec{\omega }_{ext}$ is frequently the object’s own weight) (Pollard 2004; Liu et al. 2004b). This index must be minimized to obtain an optimum grasp. As a difference with the criterion of the largest ball, this quality measure fixes the external wrench to be resisted beforehand, and then considers the required forces. The quality index is

28 $$\begin{aligned} Q_{\tiny MNF}=\min _{G\varvec{f}=\varvec{\omega }_{ext},\,M>0}\frac{1}{\sum _{i=1}^n \varvec{f}_i^n} \end{aligned}$$

with $G$ being the grasp matrix, $\varvec{f}$ the contact force vector, $\varvec{f}_i^n$ the normal component of the finger force $\varvec{f}_i$, and $M$ a matrix whose elements depend on the contact force components (Buss et al. 1995; Helmke et al. 2002). $M>0$ means that the contact forces satisfy the positivity and friction constraints (Sect. 2.1).

Another approach considers that if the forces applied at each contact point in the absence of perturbations are close to the directions normal to the object’s boundary, then the applied forces can vary in a larger range of directions to deal with external perturbations. By contrast, if the finger forces are close to the boundary of the friction cone, the fingers could easily slip when dealing with perturbations. Such quality criterion is expressed as (Han et al. 2000; Liu et al. 2004b)

29 $$\begin{aligned} Q_{\tiny DNF}=\min _{G\varvec{f}_C=\varvec{\omega }_{ext},\,M>0}\log \det M^{-1} \end{aligned}$$

**Fig. 6:**
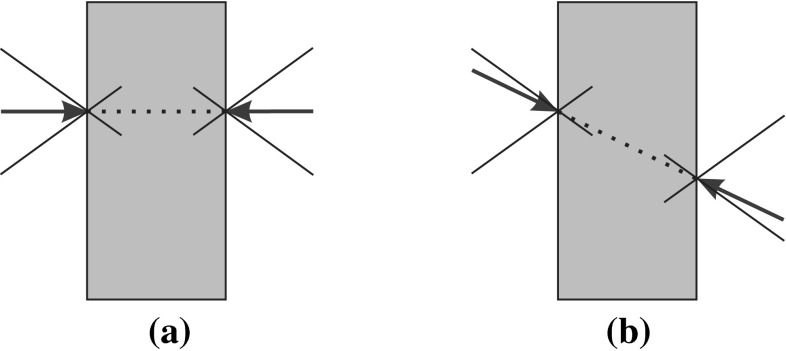
Normal components of the forces at the contact points: **a** an optimum grasp; **b** a low quality grasp

This index tends to infinity when any contact force approaches the boundary of its friction cone. Thus, smaller $Q_{\tiny DNF}$ values lead to better grasps. To illustrate this point, Fig. 6 shows 2-finger frictional grasps on a rectangle; Fig. 6a presents an example of an optimum grasp with the forces applied at the center of its corresponding friction cone, and Fig. 6b shows a low quality grasp with the forces close to the limit of the friction cone.

For 3-finger grasps on 3D objects, it is desirable that the normals at the contact points lie on the plane defined by the contact points, thus providing more room for reaction in the presence of external disturbances (Lippiello et al. 2009). Therefore, a quality index is defined as

30 $$\begin{aligned} Q_{\tiny NCP}= \frac{1}{3}\sum _{i=1}^3\left| \alpha _j-\pi /2\right| \end{aligned}$$

with $\alpha _j$ being the angle between the normal direction at the contact point $j$ and the normal to the contact plane. This index quantifies the coplanarity of the normals. Thus, lower $Q_{\tiny NCP}$ values result in better grasps.

#### Task oriented measure

An object is frequently grasped to perform a given task. When tasks are described in detail, the quality measure can quantify the ability of the grasp to counteract expected disturbances during task execution. Tasks can be characterized by a set of wrenches that must be applied on the object to achieve a desired objective, and a set of expected disturbance wrenches that the object must withstand while being manipulated. All these wrenches define a task polytope (also called *Task Wrench Space* TWS (Pollard 1996; Borst et al. 2004)), which is commonly approximated by a convex set $\mathcal{E}$ centered at the origin, such as an ellipsoid (Li and Sastry 1988) or a convex polytope (Zhu et al. 2001; Zhu and Wang 2003). The proposed quality measure is the scale factor $\lambda$ required to obtain the largest set $\lambda \mathcal{E}$ fully contained in $\mathcal{P}$. Thus, larger $\lambda$ values lead to better grasps (Borst et al. 2004; Haschke et al. 2005).

31 $$\begin{aligned} Q_{\tiny TOM}= \max _{\lambda \mathcal{E} \subset \mathcal{P},\, \lambda \ge 0} \lambda \end{aligned}$$

$Q_{\tiny TOM}$ is specifically oriented to a desired task, but in practice the constraints to be considered for some tasks may not be constant and could be difficult to define.

**Fig. 7:**
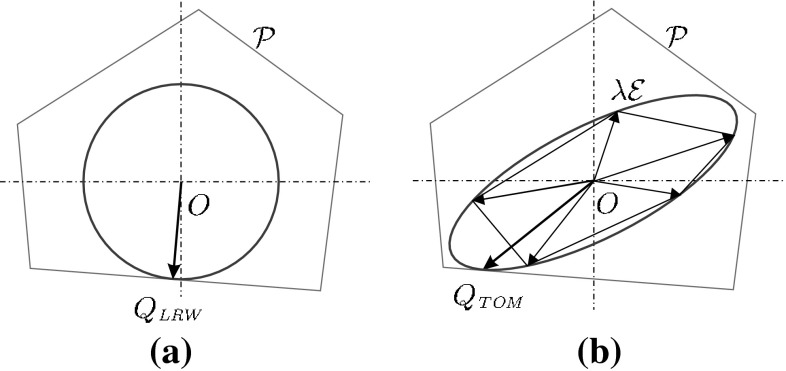
Examples of quality measures for the same applied forces: **a** Largest-minimum resisted wrench; **b** Task oriented measure

Figure 7 compares this measure (considering $\mathcal{E}$ as an ellipsoid) with the radius of the largest ball inscribed in $\mathcal{P}$. While the ball assumes that the probability for every disturbance direction is equal, the ellipsoid takes into account the most demanding wrench directions to complete the task.

By considering the set of all possible forces acting on the object surface, an approximation to the most probable perturbations on the object is obtained. In this way, the task polytope $\mathcal E$ is computed as the convex hull of the wrenches obtained by applying unitary normal forces at each contact point on a discretized object surface; tangential components of perturbation at the contact points are not included for computational reasons (Strandberg and Wahlberg 2006; Jeong and Cheong 2012).

A variation of this measure was proposed for application in interactive teaching of grasps using human examples (Aleotti and Caselli 2010). Instead of defining a task polytope, a polytope of examples $\mathcal{F}$, or *Functional Wrench Space*, is computed as the convex hull of all the primitive wrenches exerted on an object through a sequence of demonstrated grasps. A quality measure is then defined as

32 $$\begin{aligned} Q_{\tiny TBM}= \max _{\lambda \mathcal{P} \subset \mathcal{F},\, \lambda \ge 0} \lambda \end{aligned}$$

and the largest $Q_{\tiny TBM}$ indicates higher compatibility between the applied grasp and the functional set of grasps, i.e. a grasp with low $Q_{\tiny TBM}$ poorly conforms to the set of demonstrated grasps.

In unstructured environments, estimating the friction coefficient between the hand and object surface is difficult. Therefore, the minimum friction coefficient required to resist perturbations along predefined directions can as well be used as a quality measure (Mantriota 1999). A grasp configuration that minimizes this index is more robust to potential slippage of the object.

### Examples

In order to facilitate their interpretation, the measures presented above were implemented and applied to a simple 2D object, a 4 cm by 2 cm rectangle grasped with 4 frictionless fingers (unless indicated otherwise). The object contour was discretized with 64 points, 11 per each short side and 21 per each long side. For simplicity, it is assumed that a force can be punctually applied in the direction normal to a side of the rectangle, even at the vertices (in practice, a security distance must be considered). As the contacts are frictionless, each finger must lie on a different side of the rectangle, leading to 21*21*11*11=53,361 different grasp combinations, 23,100 of which are force closure grasps. For the FC grasps, different quality measures were computed. Due to the symmetric and discrete nature of the problem, several globally optimal grasps (i.e. same minimum or maximum value for different finger locations) were obtained for a given quality measure. The total number of solutions reported includes symmetric grasps due to symmetries on the finger locations.

*Measures based on algebraic properties of the grasp matrix* $G$

**Fig. 8:**
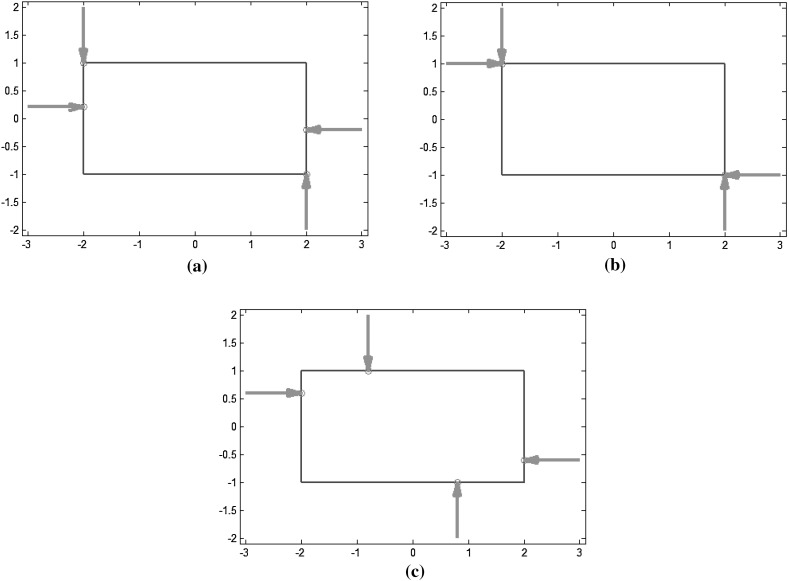
Examples of optimal grasps using different quality measures based on the properties of $G$: **a** Minimum singular value of $G$; **b** Volume of the ellipsoid in the wrench space; **c** Grasp isotropy index

- *Minimum singular value of* $G$ ($Q_{\tiny MSV}$): there are 74 optimal grasps covering different grasping options; Fig. 8a shows one of them.
- *Volume of the ellipsoid in the wrench space* ($Q_{\tiny VEW}$): there are two optimal grasps with symmetric locations of the contact points on the object; Fig. 8b shows one of them.
- *Grasp isotropy index* ($Q_{\tiny GII}$): there are four optimal grasps achieving the maximum absolute value of the quality measure; Fig. 8c shows one of them.

*Measures based on geometric relations*

**Fig. 9:**
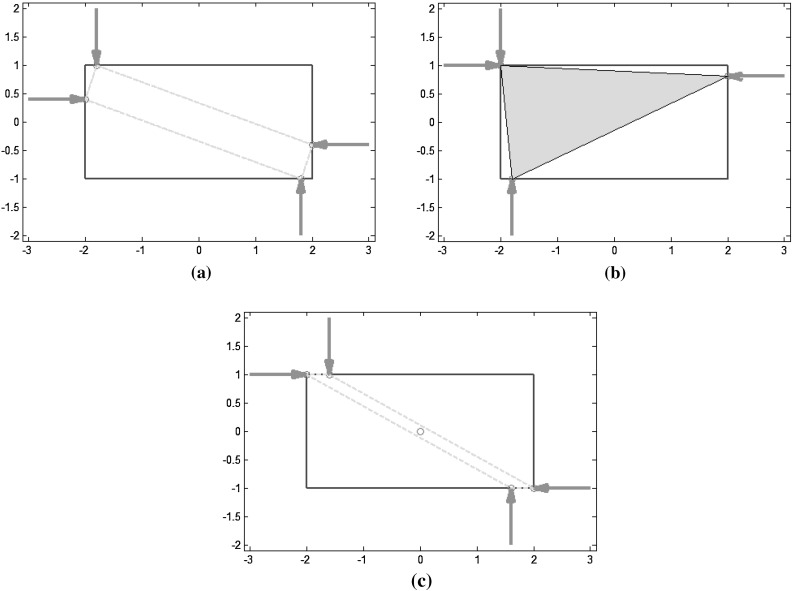
Examples of optimal grasps using different quality measures based on geometric relations: **a** Shape of the grasp polygon; **b** Area of the grasp polygon; **c** Distance between the centroid of the contact polygon and the object’s *CM*

**Fig. 10:**
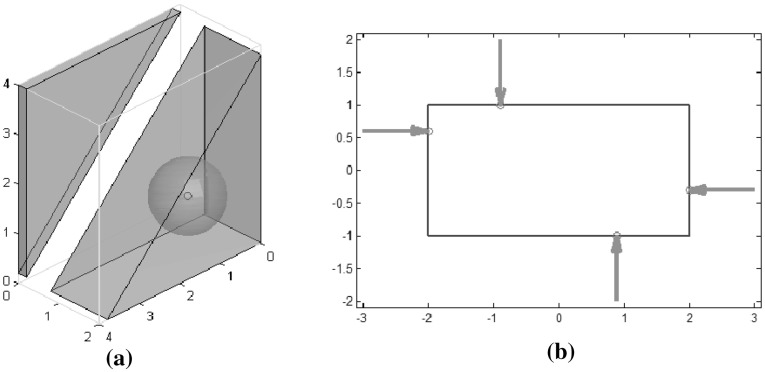
Margin of uncertainty in the finger positions: **a** Grasp space and FCS (*shaded*); **b** Optimal grasp

**Fig. 11:**
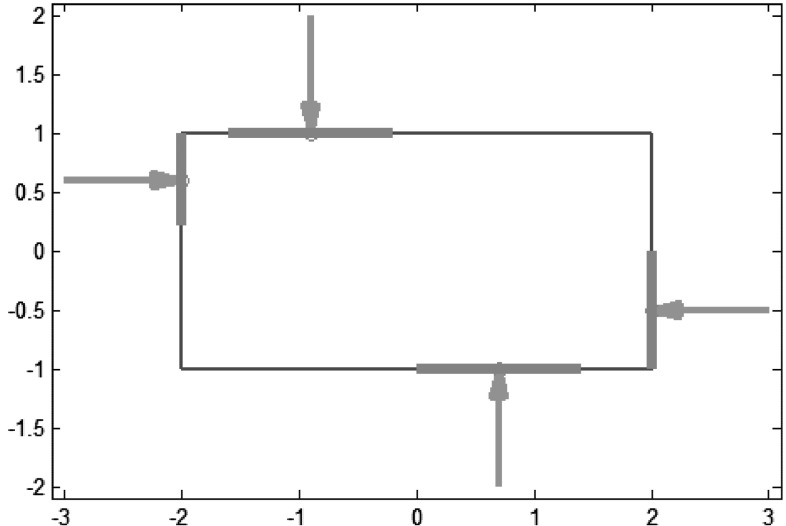
Optimal ICRs and corresponding optimal grasp according to the uncertainty grasp index

- *Shape of the grasp polygon* ($Q_{\tiny SGP}$): there are two optimal symmetric grasps on the object; Fig. 9a shows one of them.
- *Area of the grasp polygon* ($Q_{\tiny AGP}$): there are 400 different optimal grasps, with a variety of positions of the contact points on the object; Fig. 9b shows one of them.
- *Distance between the centroid of the contact polygon and the object’s center of mass* ($Q_{\tiny DCC}$): there are 100 optimal grasps that reach the minimum possible value ($Q=0$); Fig. 9c shows one of them.
- *Margin of uncertainty in finger positions* ($Q_{\tiny MUF}$): Fig. 10a shows the grasp space and force closure space (FCS) for grasps obtained when a contact point has been predefined on the rectangle; in this case, the contact on the left side of the rectangle is fixed in order to obtain a 3-dimensional representation that illustrates the concept. The largest hypersphere inscribed in the FCS determines the optimal grasp, as shown in Fig. 10b.
- *Independent contact regions* ($Q_{\tiny ICR}$): there are 4,608 optimum grasps that have the same minimum size of one of the ICRs; Fig. 11 shows one example. The figure also shows the ideal grasp according to the uncertainty grasp index for the same independent contact regions, i.e. the contact points are located in the center of their corresponding ICR.

*Measures considering limitations on the finger forces*

**Fig. 12:**
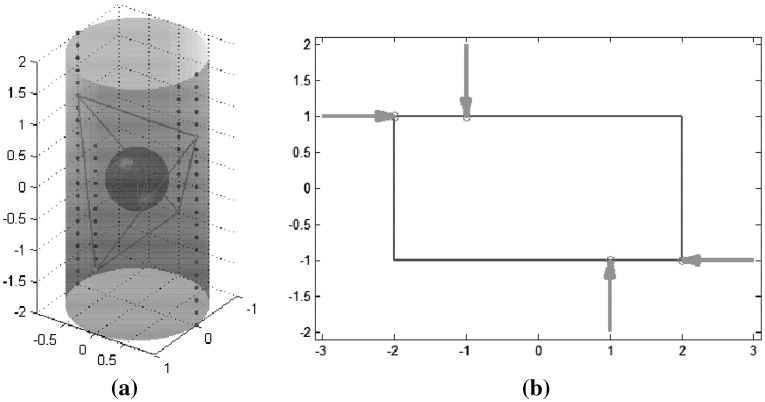
Largest minimum resisted wrench: **a** Wrench space; **b** Optimal grasp

**Fig. 13:**
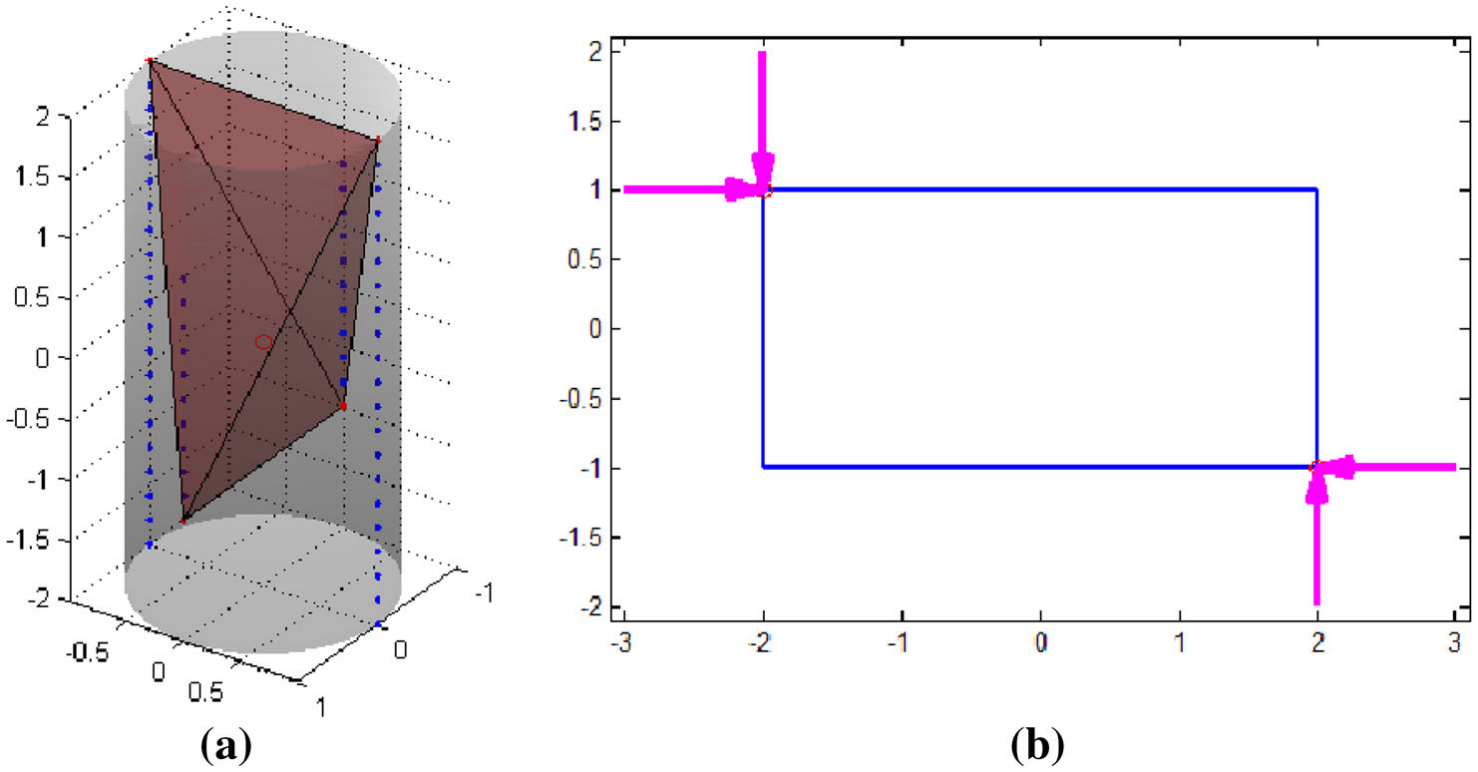
Volume of $\mathcal{P}$: **a** Wrench space; **b** Optimal grasp

**Fig. 14:**
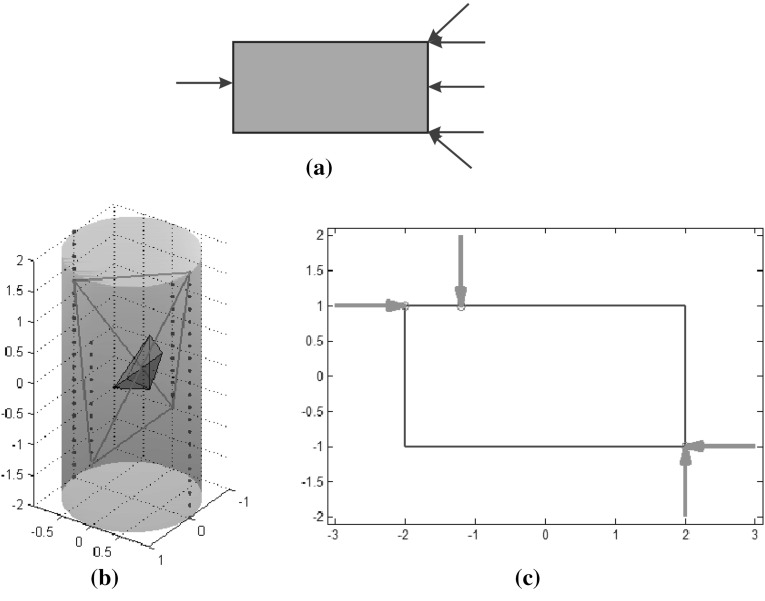
Task oriented measures: **a** Reaction forces expected in a possible contact; **b** Wrench space; **c** Optimal grasp on the object

- *Largest minimum resisted wrench* ($Q_{\tiny LRW}$): considering a limited common power source for all fingers ($\sum _{i=1}^n\left\| \varvec{f}_i\right\| \le 1$) there are two optimal symmetrical grasps; Fig. 12a shows one of them in the wrench space and Fig. 12b shows it on the object.
- *Volume of the set* $\mathcal{P}$ *of possible resultant wrenches on the object* ($Q_{\tiny VOP}$): there are two optimal symmetrical grasps; Fig. 13a shows one of them in the wrench space and Fig. 13b shows it on the object.
- *Task oriented measure* ($Q_{\tiny TOM}$): it is assumed that a task may cause the disturbances shown in Fig. 14a. There are two optimal symmetrical grasps; Fig. 14b shows one of them in the wrench space and Fig. 14c shows it on the object.

Table 1 shows a numerical comparison of the quality values for the optimal grasps according to the above criteria. Note that optimal grasps are not necessarily optimal according to all criteria. Also, different criteria may lead to similar optimal locations of the fingers on the object.

**Table 1:** Comparison of qualities for optimal grasps according to different criteria

Criterion	${Q_{\tiny MSV}}^\mathrm{a}$	${Q_{\tiny VEW}}^\mathrm{a}$	${Q_{\tiny GII}}^\mathrm{a}$	${Q_{\tiny SGP}}^\mathrm{b}$	${Q_{\tiny AGP}}^\mathrm{a}$	${Q_{\tiny DCC}}^\mathrm{b}$	${Q_{\tiny MUF}}^\mathrm{a}$	${Q_{\tiny ICR}}^\mathrm{a}$	${Q_{\tiny LRW}}^\mathrm{a}$	${Q_{\tiny VOP}}^\mathrm{a}$	${Q_{\tiny TOM}}^\mathrm{a}$
$Q_{\tiny MSV}$ (Fig. 8a)	1.4142	32.32	0.4975	0.1708	3.2	0	0.5	5	0.0828	1.4667	0.0884
$Q_{\tiny VEW}$ (Fig. 8b)	1.4142	40	0.4472	1	0	0	0.9	5	0.3162	2	0.3536
$Q_{\tiny GII}$ (Fig. 8c)	1.4142	8	1	0.4647	3.04	0	0.7	5	0.3487	0.9333	0.3333
$Q_{\tiny SGP}$ (Fig. 9a)	1.4142	27.2	0.5423	0.0199	2.56	0	0.6	5	0.1655	1.4667	0.1768
$Q_{\tiny AGP}$ (Fig. 9b)	0.0858	0.16	0.0260	0.7262	3.98	1.0512	0.9	1	0.0401	0.1333	0.0786
$Q_{\tiny DCC}$ (Fig. 9c)	1.4142	28.48	0.53	0.6772	0.8	0	0.9	5	0.3590	1.7333	0.3928
$Q_{\tiny MUF}$ (Fig. 10b)	1.1871	6.4	0.7878	0.4163	3.36	0.1	0.7	5	0.254	0.8	0.2357
$Q_{\tiny ICR}$ (Fig. 11)	1.3867	11.68	0.7957	0.3932	2.9	0.0707	0.7	5	0.2532	1.0667	0.2525
$Q_{\tiny LRW}$ (Fig. 12b)	1.4142	16	0.7071	0.6257	2	0	0.9	5	0.4472	1.3333	0.3333
$Q_{\tiny VOP}$ (Fig. 13b)	1.4142	40	0.4472	1	0	0	0.9	5	0.3162	2	0.3536
$Q_{\tiny TOM}$ (Fig. 14c)	1.3729	28.48	0.4995	0.8373	0.8	0.2	0.9	5	0.3162	1.7333	0.4419

Criteria: $^{\mathrm{a}}$maximize, $^{\mathrm{b}}$minimize

## Quality measures associated with hand configuration

This second group of quality measures includes those that consider hand configuration to estimate the grasp quality. The basic ideas from Sect. 3.1 for quality measures dependent on the properties of the matrix $G$ can be extended considering the hand-object Jacobian $H$ (Shimoga 1996), taking into account the considerations for the computation of $H$ presented in Sect. 2.2. In other cases, only hand posture (joint positions) is considered to compute a quality index.

### Measures associated with hand configuration

#### Distance to singular configurations

In order to keep redundant arms away from singular configurations, it is desirable to maximize the smallest singular value $\sigma _{min}$ of the manipulator Jacobian (Klein and Blaho 1987). The same idea is applied in grasping using the hand-object Jacobian $H$, which in a singular grasp configuration has at least one of the singular values equal to zero. Then, to be away from singular grasp configurations the index is

33 $$\begin{aligned} Q_{\tiny DSC}=\sigma _{min}(H) \end{aligned}$$

Note that $Q_{\tiny DSC}$ is conceptually equivalent to $Q_{\tiny MSV}$ given in Eq.(8), but in this case the hand-object Jacobian $H$ is considered. Therefore, it also indicates a physical condition that might be critical in a grasp from a practical point of view.

#### Volume of the manipulability ellipsoid

Analogously to $Q_{\tiny VEW}$ in Eq. (9), and in order to consider all the singular values of $H$, the volume of the manipulability ellipsoid is used as quality index (Yoshikawa 1985b). This ellipsoid is obtained by mapping with Eq. (7) a sphere of unitary radius in the velocity domain of the finger joints (i.e. the set $\parallel \varvec{\dot{\theta }} \parallel = 1$) into the object’s velocity domain, i.e.

34 $$\begin{aligned} Q_{\tiny VME}=\sqrt{\hbox {det}{\left( HH^T\right) }}=\sigma _1\sigma _2 \ldots \sigma _r \end{aligned}$$

with $\sigma _1,\sigma _2,\cdots ,\sigma _r$ being the singular values of $H$. Physically, a larger quality means that for the same velocities in the finger joints, a larger velocity of the grasped object is produced.

Note that $Q_{\tiny VME}$ is conceptually equivalent to $Q_{\tiny VEW}$ given in Eq. (9) but considering the hand-object Jacobian $H$. Therefore, it is also invariant under a change in the reference system, but does not provide information about the finger’s individual contribution.

#### Uniformity of transformation

The transformation in the velocity domain from the finger joints to the object is uniform when the contribution of each joint velocity is the same in all the components of the object velocity. In this case, the hand can move the object in any direction with the same gain, implying a good manipulation ability. The measure of this uniformity is given by the condition number of $H$ (Salisbury and Craig 1982)

35 $$\begin{aligned} Q_{\tiny UOT} = \frac{\sigma _{\max }(H)}{\sigma _{\min }(H)} \end{aligned}$$

with $\sigma _{\max }$ and $\sigma _{\min }$ being the maximum and minimum singular values of $H$.

As in the previous cases, $Q_{\tiny UOT}$ is conceptually equivalent to $Q_{\tiny GII}$ given in Eq. (10). Hence, the same reasonings about the quality properties can be applied.

#### Positions of the finger joints

A useful selection criterion with regard to poses in redundant robot arms is to find configurations whose joints are as far as possible from their physical limits, i.e. with the joint positions as close as possible to the center of their ranges (Liegeois 1977). The same idea is applied to evaluate the grasp configuration of mechanical hands. The index used to quantify joint angle deviations is

36 $$\begin{aligned} Q_{\tiny PFJ}=\sum \limits _{i=1}^{l}\left( \theta _i-\theta _{0i}\right) ^2 \end{aligned}$$

where $l$ is the total number of joints in the mechanical hand, and $\theta _i$ and $\theta _{0i}$ are the actual and middle-range positions of the $i$-th joint, respectively (the index is simplified when $\theta _{0i}=0$). $Q_{\tiny PFJ}$ could be redefined by also considering the range of each joint as

37 $$\begin{aligned} Q_{\tiny PFJ^\prime }=\sum \limits _{i=1}^{l}\left( \frac{\theta _i-\theta _{0i}}{ \theta _{\max _i}-\theta _{\min _i} } \right) ^2 \end{aligned}$$

$Q_{\tiny PFJ}$ has a simple physical interpretation and an easy computation, but even when it can produce “comfortable” hand configurations with a good range of motion for each joint, it does not necessarily imply that the hand can transmit forces or velocities in an efficient way.

The comfort of the grasp pose is even more important for humans. While defining such comfort is difficult, experiments have shown that humans prefer to use grasps where all finger joints have similar flexion values (Balasubramanian et al. 2010). Using this concept, a measure can be defined as

38 $$\begin{aligned} Q_{\tiny SFP}=\sum \limits _{i=1}^{n}\max _{j}\frac{\left| \theta _{1j}-\theta _{ij}\right| }{\theta _{\max _j}-\theta _{\min _j}} \end{aligned}$$

This measure can be relevant for humans, but not necessarily for robots, although it would certainly help to obtain more human-like hand postures.

#### Task compatibility

Consider a sphere of unitary radius in the velocity domain of the hand joints. Equation (7) maps this sphere into an ellipsoid in the generalized velocity domain (known as the velocity ellipsoid) given by

39 $$\begin{aligned} \varvec{\dot{x}}^T\left( HH^T\right) ^{-1}\varvec{\dot{x}}\le 1 \end{aligned}$$

The hand-object Jacobian $H$ was obtained in Sect. 2.2. Shimoga (1996) assumes that the same matrix can be used to express the relation between torques in the hand joint domain and wrenches in the object domain, with $\varvec{T}=H^T\varvec{\omega }$ (i.e. it is implicitly assumed that $\left\| \varvec{f}\right\| _2$ in Eq. (4) is minimized). Then, a unitary sphere in the hand joint domain can be mapped into an ellipsoid in the generalized force domain (known as the force ellipsoid) given by

40 $$\begin{aligned} \varvec{\omega }^T\left( HH^T\right) \varvec{\omega }\le 1 \end{aligned}$$

Both ellipsoids also receive the generic denomination of manipulability ellipsoids (Yoshikawa 1984). Matrices $HH^T$ and $\left( HH^T\right) ^{-1}$ are the inverse of each other, that is, they have the same eigenvalues and eigenvectors, and therefore both ellipsoids have the same volume and axes with the same directions but with lengths in inverse proportion (i.e. the direction with the maximum transmission ratio for velocities has the minimum transmission ratio for force, and vice versa). Then, the largest force and velocity gains (when applying a force on the object or giving a velocity to it) are along the direction of the major axis of the force and velocity ellipsoids, respectively, and the most accurate control of force or velocity is along the direction of the minor axis of the force or velocity ellipsoids, respectively (Chiu 1987).

If certain directions of wrenches are more likely to be applied on the object, the grasp should try to ensure the maximum wrench response along these directions (Chiu 1988). Consider a unitary vector $\varvec{\hat{\omega }}_i$ in the wrench space with the direction of a force requirement, and the distance $a_i$ from the origin to the surface of the force ellipsoid in the direction $\varvec{\hat{\omega }}_i$. Thus, $a_i\varvec{\hat{\omega }}_i$ represents a point on the force ellipsoid satisfying

41 $$\begin{aligned} \left( a_i\varvec{\hat{\omega }}_i\right) ^T\left( HH^T\right) \left( a_i\varvec{\hat{\omega }}_i\right) =1 \end{aligned}$$

from where

42 $$\begin{aligned} a_i=\left[ \varvec{\hat{\omega }}_i^T\left( HH^T\right) \varvec{\hat{\omega }}_i\right] ^{-1/2} \end{aligned}$$

Analogously, consider a unitary vector $\varvec{\hat{\xi }}_j$ with the direction of a velocity requirement, and the distance $b_j$ from the origin to the surface of the velocity ellipsoid in the direction $\varvec{\hat{\xi }}_j$. Thus, $b_j\varvec{\hat{\xi }}_j$ satisfies

43 $$\begin{aligned} \left( b_j\varvec{\hat{\xi }}_j\right) ^T\left( HH^T\right) ^{-1}\left( b_j\varvec{\hat{\xi }}_j\right) =1 \end{aligned}$$

from where

44 $$\begin{aligned} b_j=\left[ \varvec{\hat{\xi }}_j^T\left( HH^T\right) ^{-1}\varvec{\hat{\xi }}_j\right] ^{-1/2} \end{aligned}$$

With these elements, the task compatibility index is defined as

45 $$\begin{aligned} Q_{\tiny TCI}=\sum \limits _{i=1}^{s}\kappa _i a_i^{\pm 2}+\sum \limits _{j=1}^{z}\kappa _j b_j^{\pm 2} \end{aligned}$$

with $s$ and $z$ being the number of directions with, respectively, specified force and velocity requirements; the positive exponent $+2$ is used in the directions where the force or velocity magnitude should be high and the negative exponent $-2$ is used in the directions where there are requirements of precise velocity or force control, and $\kappa _i$ and $\kappa _j$ are factors to weight the relative importance of each magnitude and precision requirement.

$Q_{\tiny TCI}$ is specifically oriented to a desired task but, as for all task oriented measures, in practice the task constraints to be considered might be non-constant and difficult to define.

In some cases, the task parameters—position, forces, and velocities—can define a desired region (in the parameter space) required to achieve the task. Several grasping points and hand configurations can be considered for solving the task, and for each grasp/hand configuration a feasible region for each task parameter can be computed. Let $\lambda _f$ and $\lambda _r$ be the distances from the origin to the feasible and required sets (of forces, velocities, positions) along a given direction in the corresponding space. A safety margin is defined as

46 $$\begin{aligned} SM= {\left\{ \begin{array}{ll} \min {\frac{\lambda _f}{\lambda _r}}, &{} \text {if}\, \lambda _r\ne 0,\\ 0, &{} \text {otherwise}. \end{array}\right. } \end{aligned}$$

and the overall safety margin is the minimum value with respect to all possible directions (Sato and Yoshikawa 2011). Let $SM_p$, $SM_v$ and $SM_f$ be the safety margins for position, velocity and force at a certain grasping configuration. The quality measure is then defined as

47 $$\begin{aligned} Q_{\tiny SM}=\min \left( SM_p, SM_v, SM_f\right) \end{aligned}$$

Another way to deal with task requirements is to derive the minimum joint torques required to balance any wrench in the required force set. Given those minimum joint torques, the characteristics of the mechanical actuators restrict the joint velocities that can be applied, so a region of usable joint velocities can be defined. The maximum object velocity available in any direction is used as quality measure (Watanabe 2010)

48 $$\begin{aligned} Q_{\tiny MOV}=\min _{\varvec{\dot{x}}\in \partial \mathcal{V}}\left\| \varvec{\dot{x}}\right\| \end{aligned}$$

where $\partial \mathcal{V}$ is the boundary of the polytope describing the possible object velocities applicable to the object once the minimum torques are applied to it.

### Examples

**Fig. 15:**
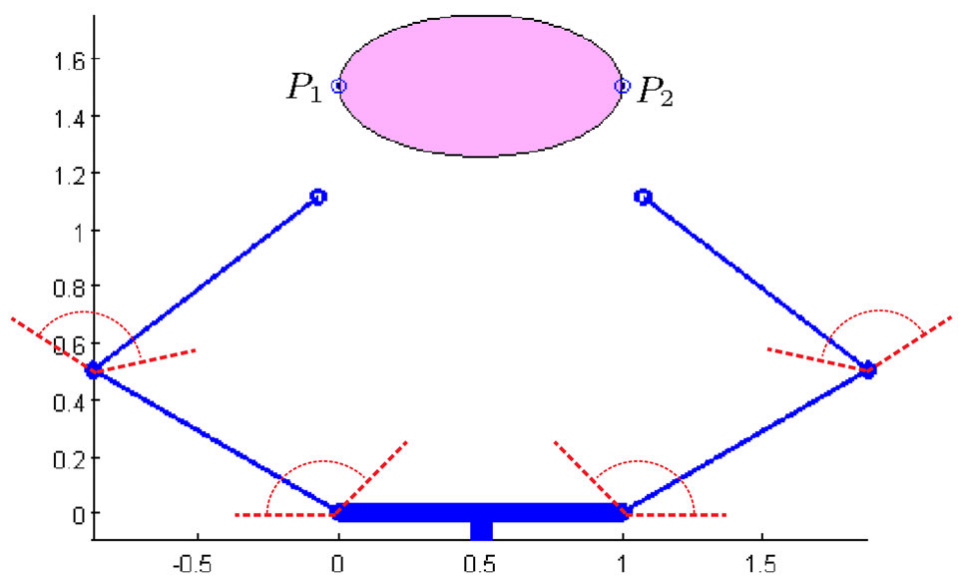
Gripper and object used in the implementation of the quality measures related to the gripper configuration

**Fig. 16:**
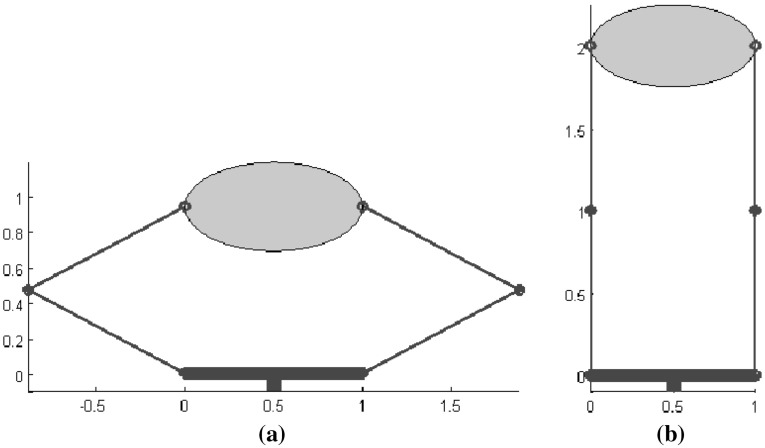
Distance to singular configurations: **a** Optimal configuration; **b** Singular configuration

**Fig. 17:**
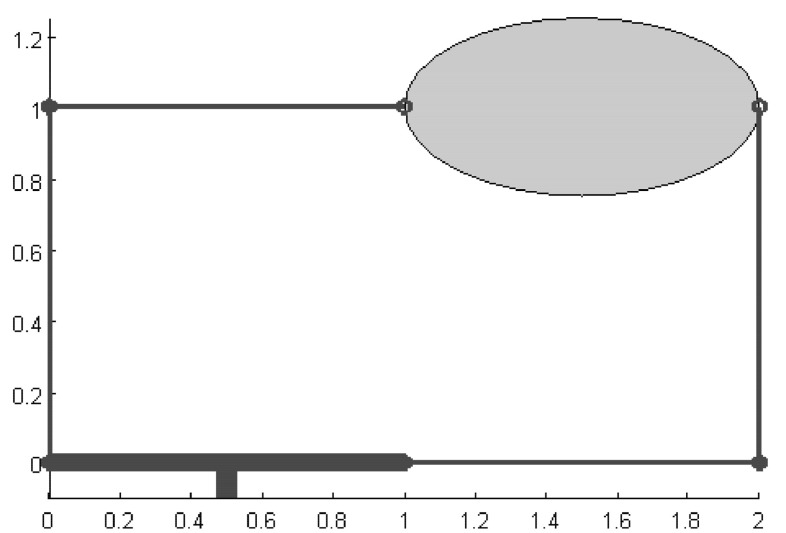
Volume of the manipulability ellipsoid: optimal configuration

**Fig. 18:**
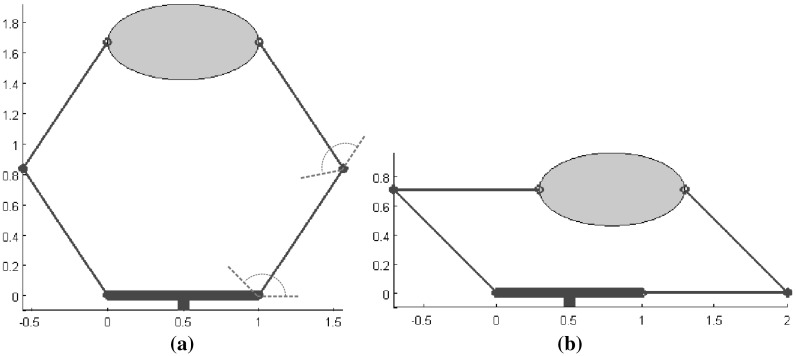
Joint angle deviations: **a** Optimal configuration; **b** Low quality configuration

Several quality measures that do not depend on a particular task were implemented for a 2-finger planar gripper. Each finger has two links and two degrees of freedom. The gripper must grasp an ellipse of 1 cm by 0.5 cm by its major axis. The gripper base and all the finger links are 1 cm long, and all joints are able to span $135^{\circ }$, as shown in Fig. 15. The workspace for the left finger has been approximated by discretizing each joint’s movement with 12 different positions. Then, for each configuration of the left finger, the configurations of the right finger that allow the grasp of the object at the predefined contact points were computed. 132 valid configurations were obtained in this way, and the following quality measures were considered:

- *Distance to singular configurations*: the optimal gripper configuration is shown in Fig. 16a. Figure 16b illustrates a singular configuration with the minimum singular value equal to zero (the worst possible quality).
- *Volume of the manipulability ellipsoid*: there are 12 optimal gripper configurations (including symmetrical poses); Fig. 17 shows one of them. These configurations allow high manipulability of the object (with respect to infinitesimal movements); however, there are joints close to their range limits.
- *Uniformity of transformation*: there are two optimal gripper configurations, which are the same as those previously obtained using the maximum distance to singular configurations (Fig. 16). The worst quality measure is also obtained in the same singular configurations. Thus, for this particular example the behavior of the two quality measures is similar.
- *Joint angle deviations*: Figure 18a shows the optimal gripper configuration, and Fig. 18b shows a low quality configuration. Note the difference between this optimal configuration, which provides more “comfort” or a larger range of possible hand movements, and the configuration in Fig. 16a, which drives the gripper away from singular configurations.

## Combinations of quality measures

Grasp quality is measured according to the above criteria, based either on the location of contact points on the object or on the hand configuration. However, the optimal grasp for some particular tasks could be a combination of these criteria; for instance, the selection of optimal contact points on the object surface according to any criteria from Sect. 3, ignoring the actual hand geometry, could lead to contact locations unreachable for the real hand, and vice versa: an optimal hand configuration could generate a weak grasp in the presence of small perturbations. Studies of correlation between quality measures show that in fact using a combination of quality measures allows capturing different aspects of prehension, like geometrical restriction, ability to resist forces, manipulability or comfort (Leon et al. 2012). To evaluate these different aspects, there have been several proposals of quality measures obtained as a combination of those presented in the previous sections, either using them in a serial or in a parallel way.

The serial approach is applied in grasp synthesis by using one of the quality criteria to generate candidate grasps, and the best candidate is chosen among them using another quality measure. For instance, the optimization with respect to the hand configuration using the weighted sum in the task compatibility index given by Eq. (45) generates a preliminary grasp. This grasp is subsequently used as initial one in the search for an optimum grasp under the measure of the largest ball given by Eq. (24) (Hester et al. 1999).

The parallel approach combines different quality measures in a single global index. A simple method uses the algebraic sum of the qualities resulting from each individual criterion (or the inverse of some criteria so that they all must be either maximized or minimized), eventually using suitable weights and normalizations. Simple addition has been used to choose optimum grasps for 2D (Boivin et al. 2004) and 3D objects (Aleotti and Caselli 2010). A variation normalizing the outcome of each criterion, dividing it by the difference between the measures of the best and the worst grasp, has been used to evaluate grasps of 2D objects performed by a 3-finger hand (Chinellato et al. 2003). Different combinations can thus be obtained by adding different basic criteria in order to generate indices specifically adapted for different practical applications (Chinellato et al. 2005).

Another approach considers a set of normalized indices and selects as quality output the minimum value among all of the normalized measures. An example of this approach uses normalized quality measures (including uncertainty in finger positions, maximum force transmission ratio, grasp isotropy and stability), assigns weights according to the desired grasp properties, and then selects the grasp with the minimum value out of the normalized and weighted measures (Kim et al. 2004).

Other possibility for combining criteria in a parallel way is to generate ranks of candidate grasps according to different quality measures, and then assign to each grasp a new index obtained as the addition of its place in each one of the original rankings. However, this approach has a high computational cost and has not provided a satisfactory outcome (Chinellato et al. 2003).

## Other criteria for quality measures

### Relation to human grasp studies

Traditional studies of human grasps have focused on aspects such as the relation between object size and hand aperture (Cuijpers et al. 2004), hand preshaping and fingertip trajectories (Supuk et al. 2005), or force distribution among fingers during object manipulation (Li 2006). Only recently has the application of concepts coming from the robotic world to the analysis of human grasps gained attention. For instance, human experience in grasping has been used to guide a robotic arm and hand to grasp objects, and lately to compare human-guided grasps to grasps obtained with a planner (Balasubramanian et al. 2010). From that work, it was evident that humans prefer to align the palm with the object’s principal axis.

More recent works have collected human grasp data with a sensorized object, and the grasps were later analyzed using different quality measures to evaluate how grasp quality increases with the number of fingers and with the contact area involved in the grasp action, to study the drawbacks of approximating a contact region with simple contact points, and to verify whether subject perception of grasp robustness matches with the prediction of the studied quality measures for both power and precision grasps (Roa et al. 2012).

Physiological aspects might be overlooked when applying pure robotic measures to analyze human grasps. Therefore, a measure that considers the biomechanical aspect in grasp evaluation is required. In (Leon et al. 2012), such index is proposed using a definition of biomechanical fatigue (Brand and Hollister 1992).

49 $$\begin{aligned} Q_{\tiny BF}=\sum \limits _{i=1}^{m}\left( \frac{F_i}{PCSA_i}\right) ^2 \end{aligned}$$

where $m$ is the number of considered muscles, $F_i$ the force exerted by each muscle (estimated with a biomechanical model of the hand), and $PCSA_i$ is the physiological area of each muscle. Smaller $Q_{\tiny BF}$ values lead to better grasps in terms of required human effort.

### Performance based measures

Existing grasp planning approaches rely mainly on quasistatic assumptions, i.e. the object does not move when the contacts are established. Causal correlation between classical quality measures such as $Q_{LRW}$ and $Q_{VOP}$ with the actual success in human grasps indicates that a high value of $Q_{LRW}$ or $Q_{VOP}$ does not necessarily imply a successful grasp in a real environment (Balasubramanian et al. 2010). The same phenomenon has recently been observed when analyzing grasp databases and comparing them with real grasp executions (Kim et al. 2013). The resulting grasp can be far from the assumed pose at planning time due to uncertainties in real systems, which results in wrong contact information and therefore wrong estimation of grasp quality. However, pose uncertainty can be considered for computing the probability of obtaining a force closure grasp (Weisz and Allen 2012). Incorporation of dynamic simulations into grasp planning systems has recently been proposed to evaluate changes in the relative pose between the hand and the object, and to predict robustness during grasping. Comparisons between simulations and real experiments have been presented for 2D (Zhang et al. 2010) and 3D cases (Kim et al. 2013).

Judging real robotic systems performing grasping actions is more challenging. For this purpose, performance-based measures are proposed to provide a score depending on the success of the system when lifting the object. A simple binary score evaluates whether the robot is able to lift the object and hold it for a predefined amount of time (Saxena et al. 2008), or whether the robot is able to hold the object even after shaking it (Balasubramanian et al. 2010; Morales et al. 2003). More elaborated discrete scoring systems can be created by considering, for instance, resistance to small perturbations directly applied on the object, deliberately trying to break the grasp (Kim et al. 2013).

After grasping the object, sometimes it changes the relative position with respect to the hand due, for instance, to dynamic effects not considered at planning time. This deviation in the object pose can also be used as an estimation of the quality of the real dynamic grasp (Kim et al. 2013):

50 $$\begin{aligned} Q_{\tiny DG}=1-\frac{\delta _{max}}{\delta _{lim}} \end{aligned}$$

with $\delta _{max}$ being the pose deviation and $\delta _{lim}$ a predefined limit for such deviation. To simplify the problem, position and orientation can be used independently to obtain the value of $Q_{\tiny DG}$, and the total score is just the minimum between position and orientation scores. The deviations in object position ($\delta _p$) and orientation ($\delta _R$) are given by

51 $$\begin{aligned} \delta _p=\left\| p_{CM}-\bar{p}_{CM}\right\| , \,\, \delta _R=\left\| log(\bar{R}^T R)\right\| \end{aligned}$$

with $p_{CM}\in \mathbb {R}^3$ being the position of the CM, $R\in SO(3)$ the orientation of the object, and the bar indicating the references for the deviations.

Performance-based indices measure the success of a grasp after its execution by lifting the object or by applying some small perturbation to it, which allows, for instance, the evaluation of the actual robustness of each grasp to store the results in a database that can be used in future grasp applications. Nevertheless, for real applications one might be interested in predicting the robustness of any grasp before actually executing it, i.e. the object should resist disturbances while being robust to uncertainties in perception and actuation, which can be tackled by using quality measures described in the previous sections.

## Discussion and conclusions

This paper has presented several grasp quality measures (summarized in Table 2) applicable to the synthesis and evaluation of fingertip grasps. The quality measures have been classified into two large groups: measures associated with the location of contact points on the object boundary, and measures associated with the hand configuration. Most quality measures in the literature are associated with the location of contact points, so this first large group was divided into three subgroups. The first one contains measures based on algebraic properties of $G$, which have limited practical application as they do not consider any restriction on the forces applied at the contact points. The second subgroup considers the measures based on geometric relations of grasp, oriented toward the improvement of grasps in the presence of inertial forces and the synthesis of independent contact regions. They are specially used to provide robustness to the grasp. The third subgroup contains measures that consider limitations on the finger forces, and includes one of the most used criterion in grasp synthesis, i.e. the largest ball and its variations.The second large group of quality measures includes criteria defined to obtain appropriate hand configurations for the grasp. A proper grasp should be optimal with respect to both groups of quality measures, and with this purpose different global quality indexes have been proposed to simultaneously quantify the grasp with respect to both groups.

**Table 2:** Grasp quality measures

Group	Subgroup	Quality index	Criterion
Measures related to the position of the contact points on the object	Based on algebraic properties of $G$	Minimum singular value of $G$	Maximize
		Volume of the ellipsoid in the wrench space	Maximize
		Grasp isotropy index	Maximize
	Based on geometric relations	Shape of the grasp polygon$^\mathrm{a}$	Minimize
		Area of the grasp polygon	Maximize
		Distance between the centroid $C$ and the center of mass *CM*	Minimize
		Orthogonality	Minimize
		Margin of uncertainty in finger positions$^\mathrm{b}$	Maximize
		Based on independent contact regions	Maximize
	Considering limitations on the finger forces	Largest-minimum resisted wrench	Maximize
		Volume of the Grasp Wrench Space	Maximize
		Decoupled forces and torques	Maximize
		Normal components of the contact forces	Minimize
		Coplanarity of the normals$^\mathrm{a}$	Minimize
		Task oriented measures	Maximize
Measures related to hand configuration		Distance to singular configurations	Maximize
		Volume of the manipulability ellipsoid	Maximize
		Uniformity of transformation	Minimize
		Finger joint positions	Minimize
		Similar flexion values	Minimize
		Task compatibility index	Maximize
		Safety margin	Maximize
Other measures		Biomechanical fatigue	Minimize
		Deviation in object pose	Minimize

$^\mathrm{a}$ Applicable only to 2D and 3D planar grasps

$^\mathrm{b}$ Applicable only to 2D grasps

Although some studies compare the optimal grasps obtained according to different criteria for different objects in 2-dimensional (Bone and Du 2001; Morales et al. 2002; Borst et al. 2004) and 3-dimensional grasps (Miller and Allen 1999), the selection of the best criterion in each real case is not always trivial. Besides, even knowing the criterion to be applied, the complexity of real cases often makes the computational cost of any grasp optimization really high. In order to provide an idea of the behavior of each quality measure, Sects. 3.4 and 4.2 present application examples on simple cases that allow the intuitive interpretation of the measure. In fact, it is not possible to provide a general recommendation for the use of any grasp quality measure, as the quality value depends on several aspects of the grasp. In general, quality measures may consider: (a) locations of the contact points on the object, (b)  directions of the forces applied at the contact points, (c)  magnitudes of the applied forces at the contact points, and (d) gripper configuration. The consideration of these elements may provide a better idea on the most convenient quality measure for a particular task.

Most of the presented grasp analysis is based on quasi-static considerations. Dynamic manipulability was originally proposed for serial manipulators (Yoshikawa 1985a, 2000), and was formulated for cooperative robots as the ratio between an input torque and the resultant acceleration of the grasped object (Bicchi et al. 1997). The concept has been recently extended to the field of multifingered grasping (Yokokohji et al. 2009).

The commercial availability of hands with integrated tactile sensors and fingertip sensors that can be adapted to specific hands (Silva et al. 2013; Yousef et al. 2011), also provides a new field of application for the presented quality measures, traditionally associated to grasp planning stages. In fact, a fingertip sensor could provide information on the magnitude of the contact force and its point of application, which can be used to estimate the direction of the force being applied on the object. This information is exploited for locally optimizing some quality index by adjusting the grasp force or even the contact location, such that the overall grasp stability during real executions is increased (Dang and Allen 2013; Laaksonen et al. 2012; Bekiroglu et al. 2011).

Some studies have analyzed the change of grasp quality with the location of contacts and the variation of the friction coefficient (Zheng and Qian 2004), and even with the number of contacts (Rosell et al. 2010). It has been suggested that, without other considerations, grasp quality increases slightly for more than a given number of contact points. A large number of contact points is typical in power grasps, but the applicability of quality measures for power grasps has hardly been tackled. One way to quantify the robustness of a power grasp is by considering the minimum virtual work rate required to move the object along a virtual displacement (Zhang et al. 1994). Another metric was proposed to minimize the distance between the object and predefined contact points on the hand, which was used to plan a pregrasp shape that is later used for online grasp planning (Ciocarlie and Allen 2009). Although in theory most of the above measures can be applied to grasps with any number of contact points (Roa et al. 2012), the explicit consideration of the limited forces that some parts of the hand can apply on the object allows the definition of contact robustness, i.e. how far a contact is from violating contact constraints, which is different from grasp robustness, i.e. how far the grasp is from overcoming the object immobilization constraint (Prattichizzo et al. 1997).

Most of the measures presented in this survey were developed for fingertip grasps using fully actuated multifingered hands. The application of the measures to underactuated hands, in particular the measures related to gripper configuration (Sect. 4), requires the development of new theoretical tools. For instance, if finger joints are modeled as elastic elements, the instantaneous kinematics of the hand and object can be predicted by considering a quasi-static equilibrium when the hand is perturbed (Quenouelle and Gosselin 2009; Odhner and Dollar 2011). Such mapping allows the application of the presented manipulability measures. The theoretical framework of parallel robots has been recently proposed as a tool for studying fingertip grasps and dexterous manipulation for underactuated hands (Borras and Dollar 2013). The adaptation of classical manipulability indices (condition number, singular values) to parallel robots has been studied and they do not seem to be consistent for analyzing such robots (Merlet 2006); the adaptation of grasp quality measures for underactuated hands is currently an open area of research (Malvezzi and Prattichizzo 2013).

The grasp quality measures reported in this survey do not consider the effect of compliance. For analyzing compliant grasps a grasp stiffness matrix $K$ is required; the grasp is stable if the stiffness matrix is positive definite (Howard and Kumar 1996). A measure of grasp stability is based on the eigenvalue decomposition of the generalized matrix $M^{-1}K$, with $M$ a metric that allows that twists and wrenches lie on the same vector space (Bruyninckx et al. 1998). However, this measure depends on the choice of the metric $M$. A frame-invariant quality measure can also be developed based on the computation of principal rotational and translational stiffnesses for a grasp with stiffness matrix $K$ (Lin et al. 2000).

When dealing with whole-hand grasps, in general it is not possible to generate forces in all directions. Thus, the concepts of active and passive force closure arise: an external wrench can be counterbalanced if there exist strictly active or passive internal forces (Bicchi and Pratichizzo 2000). Note that in this way, the condition for active force closure is stricter than for pure force closure. A grasp optimization for this case can, for instance, minimize the joint efforts (Ma et al. 2012). Also, when considering hand and contact compliance, specific solutions to the force distribution problem $\varvec{\omega }=G\varvec{f}$ can be obtained (Bicchi 1994). The implications of compliance in the grasp analysis is receiving a renewed interest due to the evolution of underactuated robotic hands (Prattichizzo et al. 2012).

There are still more open research problems related to the quality measures. First, it is worth mentioning the need for efficient algorithms (both in terms of computational complexity and computational cost) to generate optimal grasps according to different quality criteria. A second aspect is the automatic determination of the relevant quality measures for the problem at hand, either to select the most appropriate one or the most convenient combination. Even when there are already some measures that try to consider the goal of the grasp (i.e. the task to be performed), this is also an aspect that requires further research and more practical proposals. In any case, continuous advances in the development of dexterous grasping devices will require the definition and formalization of new quality measures as well as optimal procedures to apply them.

## Footnotes

1 Parameter $d$ is given by the object (2D or 3D). Restricting the analysis to force closure grasps, it is possible to obtain the minimum number of fingers required to guarantee a force closure grasp for a chosen contact model (Mishra et al. 1987; Markenscoff et al. 1990). Using this minimum number of fingers, it is verified that $d<nr$. Moreover, one of the necessary conditions for force closure is that $rank(G)=d$ (Murray et al. 1994).
